# Racial Discrimination & Cardiovascular Disease Risk: *My Body My Story* Study of 1005 US-Born Black and White Community Health Center Participants (US)

**DOI:** 10.1371/journal.pone.0077174

**Published:** 2013-10-18

**Authors:** Nancy Krieger, Pamela D. Waterman, Anna Kosheleva, Jarvis T. Chen, Kevin W. Smith, Dana R. Carney, Gary G. Bennett, David R. Williams, Gisele Thornhill, Elmer R. Freeman

**Affiliations:** 1 Department of Social and Behavioral Sciences, Harvard School of Public Health, Boston, Massachusetts, United States of America; 2 Senior Data Analyst, RTI International Waltham, Massachusetts, United States of America; 3 Haas School of Business, University of California, Berkeley, California, United States of America; 4 Psychology and Neuroscience and Duke Global Health Initiative, Duke University, Durham, North Carolina, United States of America; 5 Professor, Department of Sociology, Harvard University, Cambridge, Massachusetts, United States of America; 6 Center for Community Health Education Research and Service, Boston, Massachusetts, United States of America; Indiana University, United States of America

## Abstract

**Objectives:**

To date, limited and inconsistent evidence exists regarding racial discrimination and risk of cardiovascular disease (CVD).

**Methods:**

Cross-sectional observational study of 1005 US-born non-Hispanic black (n = 504) and white (n = 501) participants age 35–64 randomly selected from community health centers in Boston, MA (2008–2010; 82.4% response rate), using 3 racial discrimination measures: explicit self-report; implicit association test (IAT, a time reaction test for self and group as target vs. perpetrator of discrimination); and structural (Jim Crow status of state of birth, i.e. legal racial discrimination prior 1964).

**Results:**

Black and white participants both had adverse cardiovascular and socioeconomic profiles, with black participants most highly exposed to racial discrimination. Positive crude associations among black participants occurred for Jim Crow birthplace and hypertension (odds ratio (OR) 1.92, 95% confidence interval (CI) 1.28, 2.89) and for explicit self-report and the Framingham 10 year CVD risk score (beta  = 0.04; 95% CI 0.01, 0.07); among white participants, only negative crude associations existed (for IAT for self, for lower systolic blood pressure (SBP; beta  = −4.86; 95% CI −9.08, −0.64) and lower Framingham CVD score (beta  = −0.36, 95% CI −0.63, −0.08)). All of these associations were attenuated and all but the white IAT-Framingham risk score association were rendered null in analyses that controlled for lifetime socioeconomic position and additional covariates. Controlling for racial discrimination, socioeconomic position, and other covariates did not attenuate the crude black excess risk for SBP and hypertension and left unaffected the null excess risk for the Framingham CVD score.

**Conclusion:**

Despite worse exposures among the black participants, racial discrimination and socioeconomic position were not associated, in multivariable analyses, with risk of CVD. We interpret results in relation to constrained variability of exposures and outcomes and discuss implications for valid research on social determinants of health.

## Introduction

Although research on self-reported exposure to racial discrimination and health has documented consistent positive associations between higher exposure and adverse mental health, studies on somatic diseases and their risk factors are less conclusive, and have reported both positive and null findings [1]–[3], including for risk factors for cardiovascular disease (CVD), the most commonly studied somatic outcome [1]–[6]. To address these discrepancies, new work is emphasizing the need for improved measurement of exposure to racial discrimination, at multiple levels, from individual to structural, as experienced across the lifecourse and historical generation [1], [2], [7], [8]. Also garnering concern is the sociodemographic composition of study participants, given evidence that: (a) experiences and reporting of racial discrimination and its health impact may vary by lifetime socioeconomic position and nativity, and (b) “ceiling” and “floor” effects may constrain testing hypotheses, whereby if experiences of racial discrimination or of poverty are uniformly high in a given set of study participants, there may be insufficient variation to observe the postulated associations [1], [5], [7], [9], [10].

We accordingly designed the *My Body My Story* (MBMS) [11] to: (1) use a range of racial discrimination measures – spanning from individual to structural, childhood to the present-day, and explicit self-report to implicit assessment using timed reaction tests, and (2) examine their associations with risk of chronic disease in a sample of US-born black and white Americans ranging from impoverished to economically secure. In this paper, we focus on risk of CVD, analyzed in relation to 3 outcomes: systolic blood pressure (SBP), hypertension, and the Framingham 10-year CVD risk score, an integrated measure that incorporates data on age, diabetes, smoking, treated and untreated blood pressure, total cholesterol, HDL cholesterol, and body mass index (BMI) [12], [13].

Guiding our study design and analytic plan, from choice of hypotheses and study participants to selection and modeling of exposure, outcome, and covariates, is the ecosocial theory of disease distribution, which is focused on how people literally embody their societal and ecological context, at multiple levels and across the lifecourse and historical generations, thereby producing population patterns of health, including health inequities [14]–[16]. Specifically, in the case of racism and health, ecosocial theory conceptualizes racial/ethnic health inequities as biological expressions of racism and posits that there are many pathways, not just one, by which racism can harm health, with relevant pathways shaped by historical context [1], [7], [17]. These pathways can include: (a) economic and social deprivation; (b) excess exposure to toxins, hazards, and pathogens; (c) social trauma; (d) health-harming responses to discrimination; (e) targeted marketing of harmful commodities; (f) inadequate medical care; and (g) especially (but not only) for Indigenous peoples, ecosystem degradation and alienation from the land [1], [7], [17]. Additional core constructs pertain to: (1) the interplay of exposure, susceptibility, and resistance (individually and collectively), and (2) issues of accountability (causal responsibility for) and agency (the power and ability to act) at every level; a corollary is that people's bodies can tell stories that they may be unable or unwilling to articulate regarding the impact of racial discrimination upon their lives, including on their health [1], [7], [11], [18]. As ecosocial theory emphasizes, it is impossible for any given study to attempt to measure every specified pathway at every level and at all relevant spatiotemporal scales. Rather, the value of a theoretical framework is that it can help concretize systematic substantive thinking about potential causal pathways, how the selected constructs and entities are operationalized and measured, the types of statistical analyses conducted, potential threats to validity, and the complexities involved in interpreting study findings [7], [16], [19].

We accordingly used 3 approaches to measure racial discrimination, as follows.

**Figure 1:**
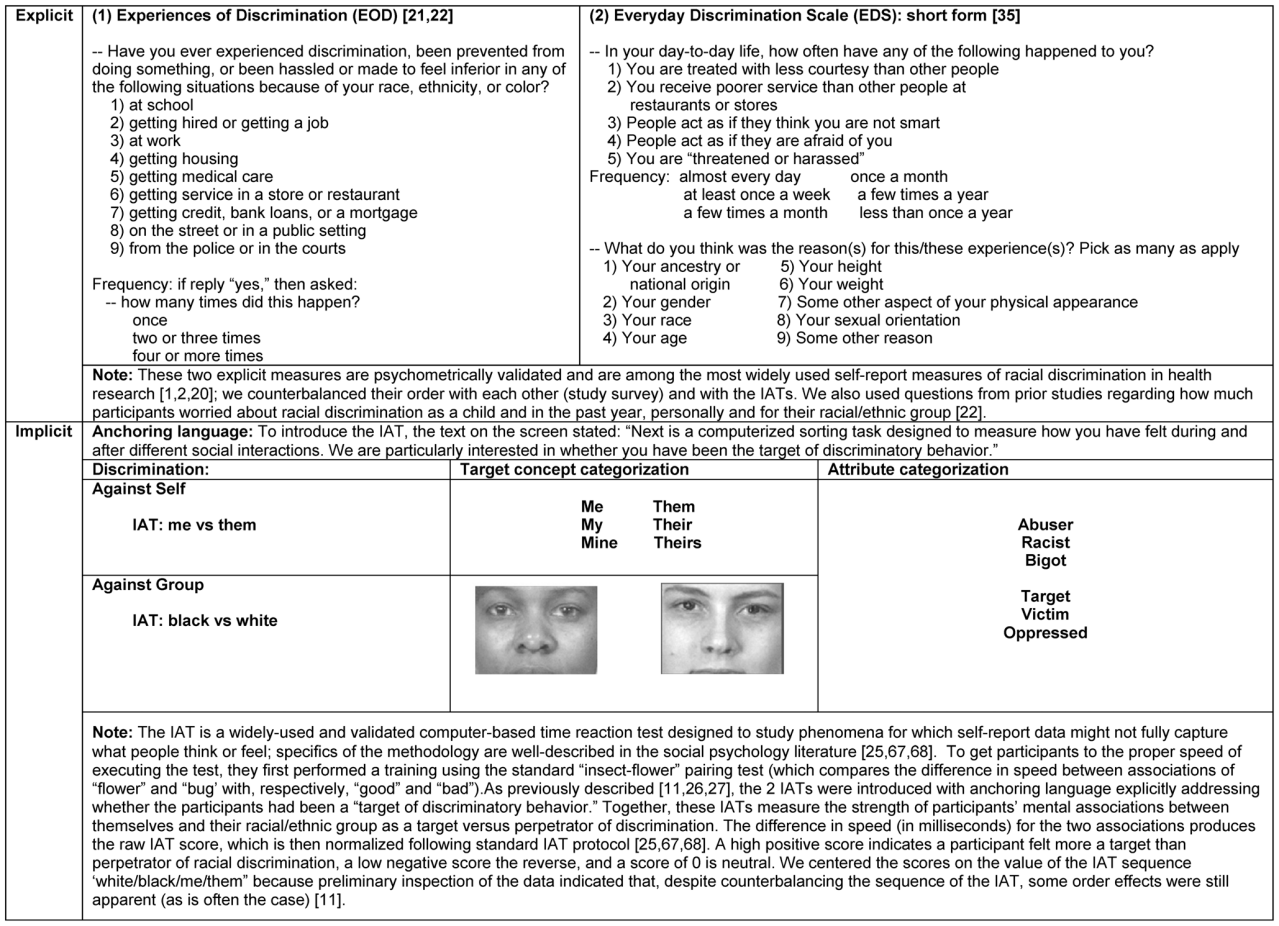
Measures of racial discrimination: implicit and explicit.

1. Explicit self-report (Figure 1), using two of the most widely-used and validated instruments [1], [2], [7], [20]: the Experiences of Discrimination (EOD) measure [21], [22] and the Everyday Discrimination Scale (EDS (any) for any unfair treatment, and EDS (race) for unfair treatment attributed to race/ethnicity) [23]. Both instruments measure both lifetime and recent exposure, but employ different approaches to obtaining data (EOD: 1-part question; EDS: 2 part question, first about experiences of unfair treatment, followed by question about attributing why, e.g., to “race”); of note, research has shown that these non-equivalent approaches to obtaining data result in different estimates of exposure and association with health outcomes [1], [7], [24].
2. Implicit Association Test (IAT) (Figure 1), a time-reaction test that measures unconscious associations, free from bias due to social concerns affecting self-report data [25]. For the implicit approach, we used a novel IAT we have formulated [11], [26], [27], the first to measure whether people unconsciously associate themselves and also their racial/ethnic group with being a target versus perpetrator of discrimination.
3. Structural, referring to population-level exposures that can shape but are not reducible to interpersonal interactions and individual experiences [1], [7]. To assess one aspect of structural discrimination, we used a new measure of Jim Crow birthplace status (i.e., born in a US state with legal racial discrimination abolished by the 1964 US Civil Rights Act) [1], [7], [28], a variable which simultaneously links levels (i.e., state and individual), lifecourse, and historical generation [1], [7].

Our *a priori* hypotheses were that: (a) each measure of exposure of racial discrimination (explicit; implicit; structural) would be associated, with increased risk of CVD, albeit not necessarily to the same extent, since each captures different aspects of exposure [1], [2], [7], [9], [25]; and (b) controlling for all 3 types of exposure to racial discrimination, along with diverse measures of socioeconomic position, would attenuate any observed black/white differences in risk of CVD, per the hypothesized pathways of embodiment leading from discrimination to health inequities involving social trauma and economic deprivation [1], [7], [16]–[18].

## Materials and Methods

As described previously [11], we recruited the 1005 MBMS participants (504 black, 501 white) from a random sample of the membership rosters of four community health centers in Boston, MA; enrollment extended from August 2008 through December 2010, a period encompassing the economic downturn linked to the 2007–2008 collapse of the US housing market and subsequent bank failures in 2008. The sample size was based on power analyses regarding likely prevalence of exposure and expected increased risk (as documented in the literature at the time of planning the study in 2007), which indicated we would have at least 80% power to detect hypothesized effect sizes ≥0.3 for high versus low exposure to racial discrimination for the specified health outcomes [11].

### Ethics statement

The study protocol, implemented in accordance with the Helsinki Declaration of 1975, as revised in 2000, was approved by the Harvard School of Public Health Office of Human Research Administration (protocol #11950–127, which covered 3 of the 4 health centers through reciprocal IRB agreements), and was also separately approved by the fourth community health center's Institutional Review Board. All participants provided written informed consent. To ensure protection of the study participants' confidentiality, the informed consent document stipulated that no data from the study may be shared without the consent of the study participants, the directors of the four community health centers from which they were recruited, and also the study's principal investigator.

### Study participants

To be eligible, persons had to self-report they were US-born, non-Hispanic, age 35 to 64, and self-identify as black or white (the preferred approach to classifying race/ethnicity for health research [1], [2], [7], [9]). Fully 94.4% of eligible screened persons agreed to participate (black: 97.0%; white: 91.9%). The overall response rate, defined as: (completed interviews)/eligible [29], equaled 82.4% (black: 86.0%; white: 81.4%) [11]. We interviewed participants at the health centers and obtained the racial discrimination, demographic, socioeconomic, psychosocial, anthropometric, biological, health status and health behavior data listed in Tables 1, 2, and 3 in five ways:

**Table 1:** *My Body My Story* study participants' sociodemographic and socioeconomic characteristics: 504 black and 501 white community health center members, all US-born, non-Hispanic, ages 35–64 (Boston, MA, 2008–2010).

Variable		Observed data		Missing: N (%)	
		Black	White	Black	White
Age (mean (SD))	years	48.6 (8.0)	49.0 (8.0)	0	0
Gender (%)	women	**69.2**	**63.1**	0	0
Poverty: (% US poverty line) (%)	below poverty line	**33.8**	**21.3**	63 (12.5)	36 (7.2)
	>100 and <200%	**21.8**	**18.5**		
	> = 200 and <400%	**19.1**	**19.1**		
	> = 400%	**25.4**	**41.1**		
Education (%)	less than high school	**16.1**	**9.9**	0	4 (0.8)
	> = high school but <4 yrs college	**68.5**	**56.5**		
	> = 4 yrs college	**15.5**	**33.6**		
Occupational class (%)	owner/self-employed/supervisor	**20.9**	**34.6**	1 (0.2)	4 (0.8)
	non-supervisory employee	**36.2**	**29.0**		
	unemployed/not in paid labor force/other	**42.9**	**36.4**		
Wealth (other than home) (%)	no financial assets	**78.7**	**54.2**	72 (14.3)	40 (8.0)
	any financial assets	**21.3**	**45.8**		
	high financial assets (> = $5,000)	**7.2**	**30.6**	88 (17.5)	63 (12.6)
Household received public assistance (%)	as a child	**52.8**	**33.2**	50 (9.9)	25 (5.0)
	in the last year	**43.6**	**28.6**	18 (3.6)	8 (1.6)
Housing tenure (%)	rent for cash	**68.2**	**46.6**	26 (5.2)	12 (2.4)
	paying mortgage/own home	**26.4**	**48.6**		
	reside without paying rent cash	**5.4**	**4.7**		
Census tract poverty (2005–2009) (%)	<5% below poverty	**6.8**	**18.5**	18 (3.6)	36 (7.2)
	5–9% below poverty	**8.4**	**27.1**		
	10–19% below poverty	**32.1**	**30.5**		
	> = 20 below poverty (“poverty area”)	**52.7**	**25.8**		
Parent/guardian born in US (%):	mother/female guardian	**94.8**	**91.2**	1 (0.2)	2 (0.4)
	father/male guardian	93.7	90.5	11 (2.2)	7 (1.4)
Parents'/guardians' education: highest attained by either parent/guardian (%):	less than high school	**27.3**	**12.0**	83 (16.5)	33 (6.7)
	> = high school but <4 yrs college	**55.1**	**51.3**		
	> = 4 yrs college	**17.6**	**36.8**		
Parents'/guardians' education: at most high school degree or GED (general equivalence diploma (%):	mother/female guardian	**70.5**	**61.3**	90 (17.9)	39 (7.8)
	father/male guardian	**72.0**	**54.9**	136 (27.0)	64 (12.8)

Note: outcomes in **bold font** have values that differ significantly (p<0.05) comparing the black versus white study participants.

**Table 2:** *My Body My Story* study participants' exposure to racial discrimination and psychosocial characteristics: 504 black and 501 white community health center members, all US-born, non-Hispanic, ages 35–64 (Boston, MA, 2008–2010).

Variable		Observed data		Missing: N (%)	
		Black	White	Black	White
**Exposure to racial discrimination**					
***Explicit (self-report)***					
Experiences of Discrimination [EOD; racial discrimination only]: ever (lifetime)				2 (0.4)	1 (0.2)
continuous (mean (SD))	number of domains or situations (range: 0–9)	**3.8 (2.7)**	**1.2 (1.7)**		
categorical (%)	0 situations	**14.1**	**50.2**		
	1–2 situations	**21.7**	**32.2**		
	3+ situations	**64.1**	**17.6**		
Everyday discrimination (EDS): in day-to-day life exposed at least 1x/year				9 (1.8)	4 (0.8)
EDS (any): continuous (mean (SD))	(range: 0–5)	**2.5 (1.6)**	**2.2 (1.5)**		
categorical (%)	unfair treatment > = 1x/yr	**86.3**	**85.1**		
EDS (race): continuous for unfair treatment due to race (mean (SD))	(range: 0–5)	**1.8 (1.8)**	**0.5 (1.3)**		
categorical (%)	unfair treatment due to race > = 1x/yr	**59.2**	**18.5**		
Worried about racial discrimination (%)	as a child, against self	**70.2**	**20.2**	1 (0.2)	2 (0.4)
	as a child, against own racial/ ethnic group	**69.8**	**30.1**	1 (0.2)	2 (0.4)
	in last year, against self	**64.0**	**20.8**	1 (0.2)	2 (0.4)
	in last year, against own racial/ ethnic group	**71.8**	**31.5**	1 (0.2)	2 (0.4)
***Implicit***					
IAT effect (mean, SD)	total Black vs White (de-trended and centered on w/b/t/m) (IAT B/W)	**0.26 (0.32)**	**0.13 (0.39)**	**10 (2.0)**	10 (2.0)
	total Me vs Them (de-trended and centered onw/b/t/m) (IAT M/T)	**0.24 (0.36)**	**0.19 (0.34)**	**11 (2.2)**	10 (2.0)
***Structural***					
Jim Crow birthplace status (%)	born in Jim Crow state	**30.2**	**5.8**	0	1 (0.2)
**Psychosocial measures**					
Response to unfair treatment (%)	take action and talk to others (act/talk)	68.2	64.3	1 (0.2)	0
	take action and keep to self (act/quiet)	9.3	10.0		
	accept as fact of life and talk to others (accept/talk)	14.7	16.4		
	accept as fact of life and keep to self (accept/quiet)	7.8	9.4		
Social desirability (mean (SD))	(range: 0–100)	**43.8 (31.7)**	**28.2 (29.5)**	27 (5.4)	11 (2.2)

Note: outcomes in **bold font** have values that differ significantly (p<0.05) comparing the black versus white study participants.

**Table 3:** *My Body My Story* study participants' health-related characteristics: 504 black and 501 white community health center members, all US-born, non-Hispanic, ages 35–64 (Boston, MA, 2008–2010).

Variable		Observed data		Missing: N (%)	
		Black	White	Black	White
***Anthropometric***					
Height (cm) (mean (SD))		167.9 (8.5)	168.3 (9.0)	0	3 (0.6)
Weight (kg) (mean (SD))		**91.2 (22.6)**	**83.3 (21.4)**	0	2 (0.4)
Body mass index (BMI; kg/m^2^) (mean(SD))		**32.4 (8.0)**	**29.3 (6.9)**	0	3 (0.6)
Obese (BMI > = 30) (%)		**57.1**	**39.4**	0	3 (0.6)
Waist circumference (cm) (mean (SD))^a^		**104.3 (15.9)**	**100.0 (16.0)**	0	4 (0.8)
Hip circumference (cm) (mean (SD))^a^		**113.0 (16.4)**	**106.0 (15.1)**	0	4 (0.8)
Waist-to-hip ratio		**0.92 (0.07)**	**0.94 (0.07)**	0	4 (0.8)
***Behavioral***					
Smoking (%)	current smoker	**43.8**	**34.5**	0	0
	ex-smoker	**16.9**	**34.5**		
	never smoker	**39.3**	**30.9**		
Physical activity (International Physical Activity Questionnaire [IPAQ]) (%)	low	**28.4**	**21.2**	1 (0.2)	0
	moderate	**30.6**	**38.3**		
	high	**41.0**	**40.5**		
Sleep: usual hours on weekdays/ workdays (%)	7 or more hours	**22.8**	**35.1**	0	0
	5 to 7 hours	**63.1**	**53.9**		
	<5 hours	**14.1**	**11.0**		
**Cardiovascular disease outcomes and related health variables**					
Blood pressure: mm Hg (mean (SD))^b^	systolic	**131.6 (16.0)**	**125.3 (16.1)**	6 (1.2)	4 (0.8)
	diastolic	**83.4 (10.7)**	**79.4 (10.4)**	6 (1.2)	4 (0.8)
Hypertension (%)	yes	**62.5**	**41.4**	8 (1.6)	6 (1.2)
Cholesterol: mg/dL (mean (SD))^b^	Total	**175.9 (35.1)**	**185.8 (36.9)**	7 (1.4)	13 (2.6)
	LDL	**103.0 (32.2)**	**112.6 (33.1)**	58 (11.5)	69 (13.8)
	HDL	**49.4 (16.0)**	**49.0 (16.6)**	23 (4.6)	19 (3.8)
	Total/HDL cholesterol ratio	**3.9 (1.6)**	**4.2 (1.7)**	27 (5.4)	23 (4.6)
Triglycerides: mg/dL (mean (SD))^b^		130.9 (84.9)	140.2 (94.9)	33 (6.5)	47 (9.4)
CVD history (self-report) (%)	yes	11.1	9.6	0	0
Diabetes (%)	yes	**25.2**	**15.1**	4 (0.8)	12 (2.4)
Framingham cardiovascular disease (CVD) 10 year risk score (mean (SD))	everyone	**10.9 (10.2)**	**9.6 (9.2)**	47 (9.3)	45 (9.0)
	only persons with no self-reported CVD history	**10.1 (9.4)**	**9.1 (9.1)**	41 (9.2)	38 (8.4)

Note: outcomes in **bold font** have values that differ significantly (p<0.05) comparing the black versus white study participants.

a % measured over clothes: (a) waist: black  = 86.1%; white  = 73.5%; (b) hip: black  = 95.6%; white  = 93.2%.

b Values for blood pressure, cholesterol, and triglycerides are reported as measured, regardless of participants' use of medication for these outcomes or their fasting status.

- (1) Audio-Computer Assisted Self-Interviewing (ACASI) [30], for the survey, administered on a computer laptop;
- (2) the IAT [11], [26], [27] (Figure 1), also administered on the laptop, with order counterbalanced with the ACASI survey (whereby participants were randomly assigned to complete the ACASI first, followed by the IAT, versus the IAT first, followed by the ACASI, in order to avoid order effects, e.g., response to IAT influenced by having taken the ACASI first, or response to ACASI influenced by having taken the IAT first);
- (3) a finger stick, to obtain blood for automated on-site analysis of the CVD biomarkers, using the validated Cholestech LDX instrument [31];
- (4) a physical exam, for the anthropometric [32] and blood pressure [33] data; and
- (5) geocoding of residential address to link to census-tract poverty level [34].

The final step of the study protocol was to debrief the participants and give them a $75 grocery card and a 26-page resource booklet (6^th^ grade literacy level) which included: (1) a two-page study debriefing statement, which briefly described the purpose of the study and the way the IAT performs as a sorting task, (2) information on their blood pressure, body measurements, and CVD biomarkers, plus text to help interpret these data and provide guidance to keep levels healthy, and (3) a resource section on organizations that provide legal assistance to address racial discrimination, and also mental health and social services [11].

### Study variables: exposures, outcomes, and covariates

In our prior methodological paper reporting on the racial discrimination, sociodemographic, and psychosocial variables [11], we provide detailed descriptions of the validated instruments employed to obtain these data. Here we briefly present the measures used, delineate how we obtained the health outcome data, and describe methods for obtaining other covariates as warranted.

#### Exposures: racial discrimination

(1) EOD [21], [22] (Figure 1). The continuous EOD measure ranged from 0 to 9 (the number of domains in which racial discrimination were ever experienced, specified in response to a 1-stage question asking about experiences of discrimination), with data also obtained on frequency of occurrence [22]. The categorical measure used cut-points based on prior research and classified participants as self-reporting no (0 domains), moderate (1–2 domains), or high (3+ domains) exposure [21], [22]. Data on response to unfair treatment was obtained using 2 yes/no questions, resulting in a 4-category typology: take action/talk to others; take action/keep quiet; accept as fact of life/talk to others; accept as fact of life/keep quiet [21], [22]. Additional questions (Figure 1) pertained to worry about racial discrimination against oneself and one's racial/ethnic group, both as a child and in the last year [22].

(2) EDS. [22], [23], [35] (Figure 1). Data were based on the short version of the EDS [35], which is a 2-part question, the first of which asks about everyday experiences of unfair treatment in 5 domains (EDS (any); scored 0–5) and their typical frequency, the second of which asks for attribution of reasons for these experiences, with 9 options provided (all of which could be checked). Persons who selected “race” or “ancestry and national” origin were classified as having experienced unfair treatment due to race/ethnicity, i.e., racial discrimination (EDS (race)).

(3) IAT (Figure 1). The two IATs were reaction-time tests regarding the associations of, respectively, self vs others, and black vs white, as target vs perpetrator of racial discrimination [11], [26], [27]. We analyzed each IAT as a continuous variable and in the analytic models also included an interaction term, to allow for synergistic effects (beyond additive) between sense of self and sense of racial/ethnic group as target vs perpetrator of racial discrimination.

(4) Jim Crow state of birth. Participants were classified as having been born in a Jim Crow state if they were born in any of the 21 states plus the District of Columbia that had legal racial discrimination overturned by the 1964 US Civil Rights Act; the 21 states were: Alabama, Arizona, Arkansas, Delaware, Florida, Georgia, Kansas, Kentucky, Louisiana, Maryland, Mississippi, Missouri, New Mexico, North Carolina, Oklahoma, South Carolina, Tennessee, Texas, Virginia, West Virginia, and Wyoming [7], [11], [28].

#### Cardiovascular outcomes

To obtain valid cardiovascular data, we asked all participants to fast for at least 8 hours prior to the exam, and all multivariable analyses controlled for whether participants had followed these instructions and also whether they had consumed food or alcohol or had smoked cigarettes 8 hours before the exam.

(1) SBP. We computed the average blood pressure based on 3 readings, taken 1 minute apart, obtained after participants had watched a 3-minute relaxation video, and as measured using a validated automatic blood pressure monitor (Omron HEM-711AC) [33].

(2) Hypertension (HBP). We defined hypertension as per the guidelines of the 7^th^ Joint National Committee on Prevention, Detection, Evaluation, and Treatment of High Blood Pressure [36], i.e., measured systolic blood pressure > = 140 mm Hg OR measured diastolic blood pressure > = 90 mm Hg OR taking blood pressure medication, with this medication history ascertained using validated questions employed in the National Health and Nutrition Examination Survey (NHANES) [37].

(3) Framingham CVD 10-year risk score [12]. We computed this score, which predicts risk (expressed as a percent) of developing cardiovascular disease in the next 10 years among persons free of CVD [12]. We ascertained if persons were free of CVD using the validated NHANES questions pertaining to “history of CVD,” referring to whether the person reported ever having been diagnosed with congestive heart failure, coronary heart disease, angina, heart attack, or stroke [37]. To create the Framingham CVD risk score, we used the gender-specific equations that incorporate data on the following variables: age, diabetes, smoking, treated and untreated blood pressure, total cholesterol, HDL cholesterol, body mass index (BMI) [12]. We obtained data on each component of the score (above and beyond blood pressure and HBP medication, described above) as follows:

- (a) Age. Self-report.
- (b) Diabetes. We followed the American Diabetes Association guidelines [38] and categorized participants as diabetic if either (i) their fasting glucose was > = 126 mg/dL, or (i) their fasting glucose was <126 mg/dL and they either were taking medicine for diabetes or self-reported having been told by a physician that they had diabetes.
- (c) Smoking. In addition to asking if participants had smoked in the 8 hours prior to the exam, we also employed validated questions to determine smoking status (current, ex, never) used in the National Health Interview Survey (NHIS) [39].
- (d) Cholesterol (total and HDL). We employed the validated Cholestech LDX instrument [31] to obtain an on-site assay of cholesterol levels, along with data on triglyceride levels.
- (e) BMI. Following NHANES protocols [32], we measured: standing height with a stadiometer (with shoes removed) to the nearest 0.635 cm (¼ inch) and weight with an electronic scale, to the nearest 0.09 kg (0.2 pounds).

#### Additional covariates

We obtained additional data on diverse sociodemographic, economic, psychosocial, anthropometric, and health behavior covariates relevant to testing the study hypotheses. In our analytic models, we retained only those variables that were associated with the exposure or outcome in at least one racial/ethnic group. Below we list the covariates we considered for analysis that did and did not meet our inclusion criteria for the analytic models; all were measured using validated instruments that are detailed in our prior methodological MBMS paper and prior analyses [11], [22].

- Sociodemographic. (i) Included: Age; gender; (ii) Not included: relationship status; parents' nativity; and racial/ethnic composition of neighborhood.
- Socioeconomic. (i) Included: poverty level; educational level (participant; participants' parents/guardians); census tract poverty; (ii) Not included: household income; occupational class; debt; wealth; receipt of public assistance; and housing tenure.
- Psychosocial. (i) Included: social desirability [40]; (ii) Not included: racial/ethnic centrality score [41]; hostility [42], [43], and occurrence of serious life event in the last year [44].
- Anthropometric. Included: waist and hip circumference with a circumference tape, to the nearest mm [32], and used to compute the waist-to-hip ratio; (ii) Not included: tibia length, measured with a flexible cloth tape, to the nearest 0.635 cm (¼ inch) [32].
- Health behavior. (i) Included: smoking (component of the Framingham risk score; see above [39]); (ii) Not included: physical activity [45]; average number of hours of sleep per night [46].

In Tables 1, 2, and 3 we report descriptive data on all retained covariates and also selected covariates not included in the analytic model that nonetheless provide relevant descriptive data about the study participants and their context.

### Statistical analyses

We conducted all analyses in SAS [47]. Guided by our theoretical framework, we assessed bivariate associations between the 3 cardiovascular outcomes (SBP; hypertension; Framingham 10-year CVD risk scores) and our *a priori* specified exposures and covariates and then retained in the analytic models only those variables empirically demonstrating statistically significant associations (p<0.05) in at least one racial/ethnic group for at least one outcome. We analyzed the Framingham risk score as an outcome, rather than its particular components (except for systolic blood pressure), because this clinically-relevant score provides a more physiologically and empirically integrated measure of cardiovascular risk than any one item by itself [12], [13]. Because analyses modeling the EOD as a categorical variable (0, 1–2, 3+) yielded no evidence of non-linear effects, we report only results for the continuous measure. We did not include the EDS (for “any” or “race”) in the analytic models because neither measure was associated with any of the outcomes.

To address the modest level of missing data (typically under 5%, except for the socioeconomic variables; see Tables 1, 2, and 3), we implemented multiple imputation via the Amelia II program [48] to create 5 imputed data sets. The imputation model included all variables retained for the analytic models (with imputations performed on component items for composite variables and re-combined in each data set), and we combined estimates across the imputed data sets using standard methods. We used linear and logistic regression, respectively, for the continuous and dichotomous outcomes; we analyzed the Framingham risk score on the log scale due to skewed distribution. We first conducted analyses separately for the black and white participants and then conducted analyses based on all participants to compare the black versus white risk of the outcome.

## Results

### Study participant profile: sociodemographic composition, exposure to racial discrimination, and health characteristics

#### Sociodemographic composition (Table 1)

All participants were, by design, US-born, and the parents/guardians' of over 90% of the black and white participants were also US-born. Both the black and white participants had adverse economic profiles, but the black participants, as previously reported [11], nevertheless were ∼1.5 times more likely to experience socioeconomic deprivation (p<0.05). For example, 33.8% of black and 21.3% of white participants were below the US poverty line; 53.7% and 23.8% respectively lived in census tracts with > = 20% below poverty (i.e., a level reaching the US census definition of “poverty area” [34]); and 84.5% and 66.4% had <4 yrs college. Despite being working age (range: 35–64; mean: 49 y), a high proportion of the black (42.9%) and white (36.4%) participants were not in the paid labor force. Moreover, only 26.4% of the black, compared to 48.6% of the white, participants were either paying mortgage on or owned their home outright, and 78.7% versus 54.2%, respectively, had no financial assets other than their home. Attesting to economic adversity across the lifecourse, over half the black participants (52.8%) and one-third of the white participants (33.2%) reported their household had received public assistance when they were a child, proportions slightly higher than those for currently receiving public assistance (43.6% and 28.6% respectively), and the highest educational level attained by their parents/guardians was, for the majority of the black and white participants, at most a high school diploma or GED (general equivalence diploma).

#### Racial discrimination (Table 2)

As also previously reported [11], the black compared to white participants were 2 to 3 times more likely (p<0. 05) to be exposed to racial discrimination (implicit, explicit, and structural). For example, fully 64.1% of the black participants, as compared to 17.6% of the white participants, reported having ever experienced racial discrimination in 3 or more domains, 70.2% v. 20.2% worried about being a target of racial discrimination as a child, and 30.2% vs. 5.8% were born in a Jim Crow state. Black compared to white participants also scored significantly higher on both IAT tests for being more likely to associate themselves and their racial/ethnic group as being a target vs. perpetrator of racial discrimination (IAT me vs them: 0.24 vs. 0.19; IAT black vs white: 0.26 vs. 0.13). Among both groups, as expected [26], [27] and also previously reported [11], the weak correlation (r<0.10) between the explicit and implicit measures was non-significant (p>0.05). Black and white participants, moreover, exhibited similar responses to unfair treatment, with 68.2% and 64.3%, respectively, taking action and talking to others, and only 7.8% and 9.4% accepting it as a fact of life and keeping it to themselves. Notably, and as previously reported [11], social desirability scores were 1.5 times higher (p<0.05) among the black compared to white participants (43.8 vs. 28.2).

#### Health outcomes (Table 3)

The black compared to white participants were significantly more likely (p<0.05), by 1.1 to 1.6 times, to have a generally poorer health profile, including for the Framingham 10-year CVD risk score (overall and also among persons free of CVD) and most of its components (BMI, systolic and diastolic blood pressure, current smoking, treated and untreated hypertension) and also for waist and hip circumference, obesity, low physical activity, and inadequate sleep. Their profiles were similar, however, for HDL cholesterol and self-reported histories of CVD, and significantly better (p<0.05) for waist-to-hip ratio, total and LDL cholesterol, and total/HDL cholesterol ratio. Overall, 57.1% of the black participants and 39.4% of the white participants were obese, 62.5% and 41.4% were hypertensive, their mean SBP (for all persons, whether or not taking anti-hypertensive medication) respectively equaled 131.6 and 125.3 mm Hg, and among persons free of CVD, their risk of developing CVD in the next 10 years was, respectively, 10.1% and 9.1%.

### Analytic results (statistical models)

Among the racial discrimination measures, statistically significant positive crude associations (95% CI excluded 0 for betas from the linear regression models and 1 for the odds ratio from the logistic regression models) occurred, among black participants only, between: (1) the Jim Crow measure and hypertension, uncontrolled hypertension, and the two Framingham scores (Table 4 and Table 5) and (2) the EOD and the Framingham CVD risk score (Table 5). Among the white participants only, a statistically significant inverse crude association occurred between the IAT me vs. them and both SBP and the Framingham CVD risk score (Table 4 and Table 5). Additionally, with regard to the socioeconomic variables, statistically significant crude positive associations for hypertension (Table 4) and Framingham CVD risk score (Table 5) existed with low parental/guardian education (black participants) and low participant education (both black and white participants). A crude positive association also tended to exist, for black participants, between poverty and hypertension.

**Table 4:** Univariate and multivariable associations of racial discrimination and socioeconomic position with systolic blood pressure and hypertension: *My Body My Story* study (504 black, 501 white US born non-Hispanic participants; Boston, 2009–2010) (imputed data).

Variable	Systolic blood pressure (mm Hg)				Hypertension			
	β (95% CI)				OR (95% CI)			
	Black		White		Black		White	
	Univariate	Multivariable	Univariate	Multivariable	Univariate	Multivariable	Univariate	Multivariable
***Racial discrimination***								
**Explicit**								
EOD: continuous (0–9)	−0.21 (−0.74, 0.32)	−0.31 (−0.87, 0.24)	−0.09 (−0.92, 0.73)	−0.38 (−1.14, 0.39)	0.96 (0.90. 1.02)	0.94 (0.86, 1.02)	1.04 (0.93, 1.15)	1.02 (0.90, 1.16)
**Implicit**								
IAT: black vs. white (B/W) as target	0.40 (−4.00, 4.81)	1.43 (−4.05, 6.90)	−1.12 (−4.82, 2.57)	−3.40 (−7.27, 0.47)	1.00 (0.57, 1.73)	0.86 (0.39, 1.90)	1.03 (0.64, 1.65)	1.04 (0.54, 1.99)
IAT: me vs. them (M/T) as target	−1.96 (−5.86, 1.93)	0.72 (−4.49, 5.92)	*−4.86 (−9.08, −0.64)*	−3.26 (−7.24, 0.72)	0.75 (0.46, 1.25)	1.03 (0.47, 2.27)	0.77 (0.45, 1.32)	0.82 (0.42, 1.62)
Interaction: IAT B/W x IAT M/T	n/a	−3.51 (−15.7, 8.67)	n/a	7.22 (−3.14, 17.58)	n/a	1.05 (0.17, 6.51)	n/a	1.49 (0.30, 7.57)
**Structural**								
born in Jim Crow state: yes vs. no (ref)	2.18 (−0.86, 5.23)	−1.26 (−4.66, 2.15)	2.85 (−3.16, 8.87)	2.76 (−2.73, 8.24)	*1.92 (1.28, 2.89)*	0.97 (0.59, 1.60)	0.62 (0.27, 1.44)	0.64 (0.25, 1.69)
*Economic*								
Poverty:	1.13 (−1.76, 4.03)	0.84 (−2.10, 3.77)	−0.17 (−3.09, 2.75)	−0.00 (−2.70, 2.69)	1.44 (0.97, 2.13)	1.50 (0.92, 2.45)	0.90 (0.61, 1.32)	0.69 (0.43, 1.10)
<200% vs.								
> = 200% (ref)								
Education:	−0.12 (−5.16, 4.92)	−1.74 (−7.23, 3.75)	4.03 (−1.13, 9.19)	1.00 (−4.25, 6.26)	1.72 (0.87, 3.38)	0.93 (0.40, 2.12)	*1.96 (1.02, 3.75)*	0.96 (0.41, 2.26)
< high school (HS)								
> = HS and <4 yrs college	−1.28 (−5.23, 2.67)	−0.46 (−4.42, 3.50)	2.74 (−0.32, 5.80)	0.91 (−2.34, 4.17)	0.79 (0.48, 1.31)	0.64 (0.35, 1.16)	1.45 (0.97, 2.18)	0.95 (0.55, 1.66)
> = 4 yrs coll (ref)	0.0	0.0	0.0	0.0	1.0	1.0	1.0	1.0
Census tract poverty: > = 20%	−1.08 (−7.07, 4.91)	−1.27 (−6.90, 4.36)	−1.08 (−5.39, 3.23)	−2.45 (-6.33, 1.42)	1.39 (0.69, 2.80)	1.35 (0.61, 3.01)	1.40 (0.77, 2.56)	1.19 (0.58, 2.45)
> = 5% and <20%	−1.40 (−7.34, 4.55)	−1.23 (−6.88, 4.42)	−2.16 (−5.99, 1.68)	−2.14 (−5.58, 1.31)	1.27 (0.62, 2.59)	1.13 (0.49, 2.59)	1.25 (0.74, 2.11)	1.42 (0.76, 2.65)
<5% (ref)	0.0	0.0	0.0	0.0	1.0	1.0	1.0	1.0
Parents'/guardians' highest education: <HS	1.04 (−5.34, 7.41)	−0.19 (−6.78, 6.40)	1.99 (−3.33, 7.30)	1.23 (−3.38, 5.83)	*1.96 (1.02, 3.77)*	1.49 (0.75, 2.97)	1.98 (0.98, 4.01)	1.99 (0.80, 4.94)
> = HS and <4 yrs college	−1.26 (−6.55, 4.03)	−1.17 (−5.92, 3.57)	1.86 (−1.66, 5.39)	1.93 (−1.22, 5.09)	1.08 (0.58, 2.01)	1.19 (0.68, 2.08)	1.40 (0.92, 2.14)	1.38 (0.83, 2.28)
> = 4 yrs coll (ref)	0.0	0.0	0.0	0.0	1.0	0.0	1.0	1.0

**Note:** Parameter estimates whose 95% CI exclude 0 for systolic blood pressure and exclude 1 for the hypertension outcomes are in ***bold highlight.*** Multivariable analyses controlled for all variables listed in the above columns and also: response to unfair treatment; social desirability; body mass index; waist to hip ratio; cigarette smoking (current and smoked within 8 hrs of exam, current did not smoke within 8 hrs of exam; ex-smoker, never smoker); alcohol within 8 hrs of exam (yes; no); food within 8 hrs of exam (yes; no); taking anti-hypertensive medication (yes; no).

**Table 5:** Univariate and multivariable associations of racial discrimination and socioeconomic position with Framingham 10-year cardiovascular risk scores: *My Body My Story* study (504 black, 501 white US born non-Hispanic participants; Boston, 2009–2010) (imputed data).

Variable	Framingham CVD 10-yr risk score (log-transformed values)			
	β (95% CI)			
	Black		White	
	Univariate	Multivariable	Univariate	Multivariable
***Racial discrimination***				
**Explicit**				
EOD: continuous (0–9)	***0.04 (0.01, 0.07*** *)*	−0.01 (−0.03, 0.01)	0.03 (−0.02, 0.08)	0.00 (−0.03, 0.03)
**Implicit**				
IAT: black vs. white (B/W) as target	−0.09 (−0.38, 0.20)	−0.17 (−0.38, 0.05)	−0.02 (−0.26, 0.21)	−0.01 (−0.17, 0.14)
IAT: me vs. them (M/T) as target	−0.20 (−0.45, 0.06)	−0.03 (−0.24, 0.19)	**−** ***0.36 (*** **−** ***0.63,*** ** −** ***0.08)***	**−** ***0.17 (*** **−** ***0.33,*** ** −** ***0.01)***
Interaction: IAT B/W x IAT M/T		0.15 (−0.35, 0.65)		−0.13 (−0.52, 0.27)
**Structural**				
born in Jim Crow state: yes vs. no (ref)	***0.57 (0.38, 0.76)***	−0.08 (−0.22, 0.06)	−0.08 (−0.49, 0.33)	0.22 (−0.01, 0.44)
***Economic***				
Poverty:	0.05 (−0.13, 0.24)	0.02 (−0.10, 0.15)	0.11 (−0.08, 0.30)	−0.04 (−0.15, 0.07)
<200% vs. > = 200% (ref)				
Education: < high school (HS)	***0.33 (0.01, 0.65)***	−0.08 (−0.29, 0.14)	***0.74 (0.41, 1.07)***	0.11 (−0.11, 0.32)
> = HS and <4 yrs college	0.02 (−0.23, 0.27)	−0.07 (−0.23, 0.08)	***0.52 (0.32, 0.71)***	***0.18 (0.05, 0.30)***
> = 4 yrs coll (ref)	0.0	0.0	0.0	0.0
Census tract poverty: > = 20%	0.13 (−0.24, 0.51)	−0.02 (−0.22, 0.19)	0.20 (−0.10, 0.49)	0.06 (−0.10, 0.23)
> = 5% and <20%	0.07 (−0.31, 0.44)	−0.06 (−0.28, 0.16)	−0.00 (−0.26, 0.25)	0.01 (−0.13, 0.15)
<5% (ref)	0.0	0.0	0.0	0.0
Parents'/guardians' highest education: <HS	***0.33 (0.00, 0.65)***	0.09 (−0.13, 0.32)	0.28 (−0.09, 0.66)	0.01 (−0.18, 0.21)
> = HS and <4 yrs college	−0.04 (−0.38, 0.30)	0.04 (−0.12, 0.21)	0.21 (−0.03, 0.44)	0.06 (−0.06, 0.18)
> = 4 yrs coll (ref)	0.0	0.0	0.0	0.0

**Note:** Parameter estimates whose 95% CI exclude 0 are indicated in ***bold highlight***. Multivariable analyses controlled for all variables listed in the above columns and also: response to unfair treatment; social desirability; body mass index; waist to hip ratio; cigarette smoking (current and smoked within 8 hrs of exam, current did not smoke within 8 hrs of exam; ex-smoker, never smoker); alcohol within 8 hrs of exam (yes; no); food within 8 hrs of exam (yes; no); taking anti-hypertensive medication (yes; no).

In the multivariable models (Table 4 and Table 5; see Table S1 for parameter estimates for the additional variables controlled for in the analyses), the sole racial discrimination measure among black participants that displayed a statistically significant association occurred in the analyses for uncontrolled hypertension, whereby the odds ratio (OR) for the IAT for me vs. them (comparing risk for persons more vs. less likely to associate themselves personally, versus others, as being a target of racial discrimination) equaled 2.85 (95% confidence interval (CI) 1.05, 7.75). In these multivariable models, none of the measures of socioeconomic position remained significant among the black participants.

Among white participants, only one measure of racial discrimination exhibited any association with any of the health outcomes: the IAT me vs them, which showed a weak inverse association with the Framingham 10-year CVD risk score (β on the log scale: −0.17, 95% CI −0.33, −0.01) (Table 5). The sole socioeconomic measure that exhibited any statistically significant association was participants' education, whereby risk was slightly elevated for the Framingham CVD risk score among persons who had at least a high school education but less than 4 years of college, as compared to persons with 4 years of college or more (Table 5).

Lastly, regarding the extent to which control for racial discrimination and socioeconomic position attenuated the observed crude black excess risk for the 3 study outcomes (SBP, hypertension, Framingham CVD risk score), results indicated that, first, following adjustment for age and gender, the black/white differences remained statistically significant for only two outcomes (Table 6, Model 1): SBP (average excess: 3.79 mm Hg, 95% CI 1.81, 5.76) and hypertension (odds ratio: 2.40, 95% CI, 1.77, 3.24). Additional control for racial discrimination (Model 2) and socioeconomic position (Model 3), separately or together (Model 4), each in conjunction with other core covariates, did not eliminate the black excess risk for either SBP or hypertension.

**Table 6:** Black vs. white difference: effect of adjusting for racial discrimination (RD)^a^ and socioeconomic position (SEP)^b^, separately and jointly (imputed data).

CVD outcome	Parameter estimate: for black vs. white	Model 1	Model 2	Model 3	Model 4
adjusting for:		(age + gender)	(RD^a^ + core^c^)	(SEP^b^ + core^c^)	(RD^a^ + SEP^b^ + core^c^)
**SBP**	β (95% CI)	***3.79 (1.81, 5.78)***	***3.77 (0.23, 7.30)***	***3.81 (1.63, 5.98)***	***3.80 (0.12, 7.48)***
**Hypertension**	OR (95% CI)	***2.40 (1.77, 3.24)***	***2.96 (1.72, 5.12)***	***2.14 (1.55, 2.97)***	***2.70 (1.53, 4.75)***
**Framingham 10 year CVD risk score (log-transformed values)**	β (95% CI)	−0.02 (−0.10, 0.06)	0.04 (−0.10, 0.18)	−0.06 (−0.14, 0.03)	−0.00 (−0.15, 0.14)
**Model adjusts for:**					
age + gender		**✓**	**✓**	**✓**	**✓**
racial discrimination^a^			**✓**		**✓**
socioeconomic position^b^				**✓**	**✓**
core covariates^c^			**✓**	**✓**	**✓**

**Note:** models run (on the imputed data) for the total study population (504 black and 501 white participants), except for analyses for uncontrolled hypertension, which were restricted to participants with hypertension (315 black, 209 white); for estimating the black vs. white risk of the outcome, β  =  parameter estimate for linear regression, OR  =  odds ratio for logistic regression, CI  =  confidence interval; ***bold italic*** **:** 95% CI for β excludes 1 or 95% CI for OR excludes 1. Values for the Framingham scores are log transformed.

a **RD models:** included interaction terms between race/ethnicity and each RD measure (explicit: EOD; implicit: IAT/black vs. white, IAT/me vs. them, and their interaction; structural: Jim Crow birthplace status); because none of the interaction terms were statistically significant, we report only the main effect.

b **Socioeconomic position:** poverty, educational level (participant and participant's parents/guardians).

c **Core covariates**: age; gender; socioeconomic position (poverty level: household and census tract, education: respondent and highest level attained by parents'/guardians); psychosocial (response to unfair treatment, social desirability); anthropometric (body mass index, waist-to-hip ratio); health behavior (smoking status; cigarette, food, or alcohol within 8 hrs of exam); for SPB only: taking anti-hypertensive medication.

## Discussion

Our central findings were that within a random sample of working age US-born black and white American members of 4 urban health centers who overall had a poor socioeconomic and cardiovascular health profile, black participants were more likely than white participants (p<0.05) to be impoverished (33.8% vs. 21.3%), to self-report exposure to racial discrimination (in 3 or more situations: 64.1% vs 17.6%, including as a child: 70.2% vs 20.2%), to associate their racial/ethnic group and themselves with being a target vs perpetrator of racial discrimination (IAT effect: 0.26 vs. 0.13 and 0.24 vs. 0.19, respectively), and to have been born in a Jim Crow state (30.2% vs. 5.8%). Nevertheless, despite markedly higher levels of exposure to racial discrimination and impoverishment among the black compared to white participants, crude associations of racial discrimination and risk of CVD among the black participants were not robust to control for socioeconomic position and other covariates, nor did controlling for exposure to racial discrimination and socioeconomic adversity account for the black excess risk for elevated SBP and risk of hypertension or affect the null excess risk (after controlling for age and gender) for the Framingham CVD score.

### Study limitations and strengths: considering the MBMS participants in context

As a first step in interpreting our study findings, it is important to consider study limitations and strengths. One limitation is that our investigation, by virtue of study design, was cross-sectional, limiting causal inference, even as we did obtain data on exposure to racial discrimination and socioeconomic position across the lifecourse. Strengths include: the high response rate within our random sample; little missing data; and reliance on both validated instruments and innovative use of new implicit and structural racial discrimination measures.

A second limitation pertains to the unexpectedly poor and constrained socioeconomic and health profile of both the black and white participants. Here we note that we chose to recruit participants from community health centers [11] for two important reasons. First, the mandate of community health centers is to provide health care to persons who are low income and medically underserved, as well as are diverse in their racial/ethnic composition [49], and so are likely to serve populations who are harmed by racial discrimination and economic deprivation. Second, community health centers are trusted community-based organizations [49], which is especially important for recruiting participants from social groups that have been subjected to social and economic deprivation and historically exposed to unethical research practices [50]; the high MBMS participation (black: 97.0%; white: 91.9%) and response rates (black: 86.0%; white: 81.4%) attest to the value of this recruitment strategy [11]. A corollary is that community health centers are used by individuals who value these aspects of community health centers, whether or not low income, thereby resulting in an economically heterogeneous (and not solely low income) membership [49].

**Figure 2:**
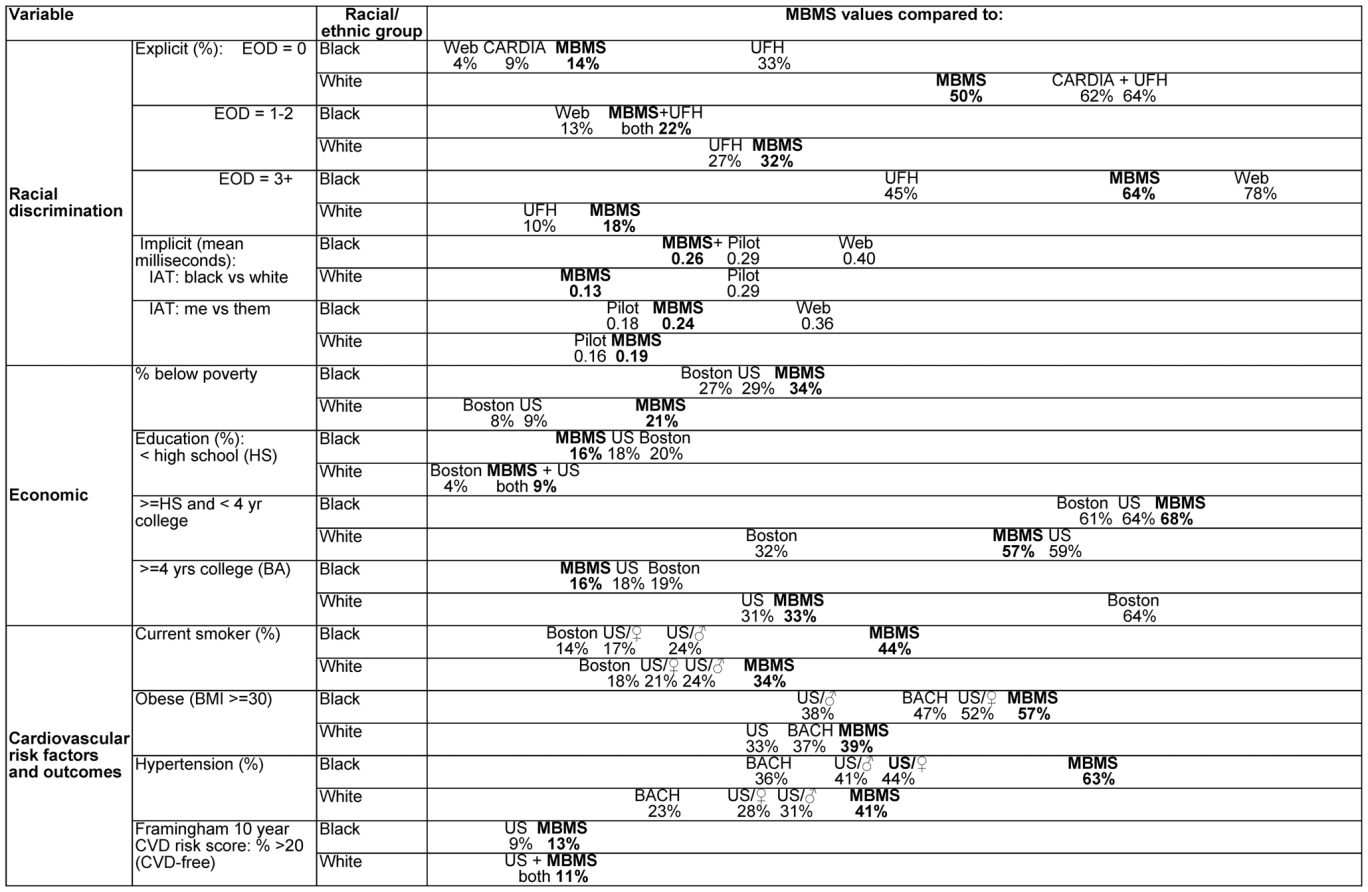
Distributions of average exposure to racial discrimination, socioeconomic position, and cardiovascular risk factors and outcomes, for My Body My Story participants (2008–2010) compared to analogous Boston data and US national data.

Thus, as expected, the MBMS study participants both black and white, spanned from those with lower to higher income and education, whereby although 33.8% of the black and 21.3% of the white participants were below the US poverty line and 16.1% and 9.9% had less than a high school education, 20% of each group nevertheless had household incomes over 400% of the US poverty line, and 15.5% and 33.6% had 4 or more years of college. Of note, and as shown in Figure 2, whereas the poverty and education levels of the black participants were on par with or worse than those of the 2010 black population in Boston and the US [51]–[53], the white participants, by contrast, were 2 to 3 times more likely to be currently impoverished compared to the 2010 white non-Hispanic population in Boston and the US [51]–[53] and half as likely as Boston's 2010 white non-Hispanic population to have completed 4 years of college [53].

Also of note, and also shown in Figure 2, the self-report and implicit measures of racial discrimination, were, for the black participants, in the lower range, and, for the white participants, in the higher range, compared to what has been reported in prior research, especially in relation to studies whose participants had higher income or education [26], [27], [54], [55]. Thus, the values for the explicit and implicit measures of racial discrimination among the black participants were similar to those of other economically deprived black populations [22], [26], but lower than those of black populations with higher education [27], [54]. By contrast, as compared to other low-income white populations, the white participants had higher values for the explicit racial discrimination measures [22], [55] and lower values for the IAT/black vs. white [26].

Perhaps most germane to interpreting results, both the black and white participants had health profiles considerably worse than their Boston and US counterparts (Figure 2). For example, the proportion of black and white MBMS participants who were current smokers, obese and hypertensive was, contrary to what we expected, 1.5 to 2 times higher compared to population-based estimates for Boston and nationally [51], [52], [56]. These comparative data thus suggest that the community health center members – from whom the MBMS participants were randomly selected, *without regard to their health status or health care utilization* – were disproportionately in poorer health compared to other persons in the same income range.

### Interpretation of results

Our effectively null results regarding associations between exposure to racial discrimination and risk of cardiovascular disease among black Americans are not an unusual finding. A 2003 review article that reviewed 17 US studies published between 1972 and 2001 observed that “The existing data on the relationship of racism to BP [blood pressure] level or HT [hypertension] status are mixed” and concluded that “methodological limitations limit their interpretability and are likely to account for the inconsistent and relatively weak findings ” (see [57], p. 62). Of note, similar summary comments can be found in a series of 3 review articles published between 2011 and 2013 focused on racial discrimination and CVD. Thus, a 2011 review article that analyzed 24 US studies published between 1984 and 2010 concluded: “Direct evidence linking individual/interpersonal racism to [hypertension] HTN diagnosis is weak” but, suggesting that acute rather than chronic effects may be more readily observed, further stated “the relationship of individual/interpersonal racism to ambulatory blood pressure (ABP) is more consistent, with all published studies reporting a positive relationship of interpersonal racism to ABP” (see [4], p. 518). A 2012 review article that critically assessed 15 studies of African Americans published between 1990 and 2012 likewise averred that although its “systematic review supports the association of racial discrimination with an increased risk of developing hypertension; however, the picture is not uniform” and argued that “[m]ethodological challenges, such as floor or ceiling effects of reported discrimination and low sample size, may have prevented researchers from detecting important associations” (see [5], p. 422). Another review article, also published in 2012, in turn focused on 22 US articles on racial discrimination and cardiovascular disease published between 2000 and 2010 and reported that, among 50 tests for associations performed, 40% were null, 30% “revealed global positive associations, of which 67% were statistically significant,” and 15% were negative (i.e., higher exposure to racial discrimination associated with lower blood pressure/hypertension)” (see [6], p. 956). Thus, conclusive evidence of either a positive or null association remains elusive.

To this mixed literature, our study contributes two methodological refinements potentially relevant to future research – i.e., the use of the IAT and the structural measure of racial discrimination. Moreover, even though our findings are not in accord with our *a priori* hypotheses, we do not deem these effectively null findings to be conclusive. Rather, in light of the above evidence we present on study participant characteristics, we argue instead that our finding of weak or no associations between risk of CVD and both the racial discrimination and socioeconomic measures likely reflects the unexpectedly poor health profiles of the MBMS participants, as well as the high levels of lifetime socioeconomic adversity among both the black and white participants. The net impact, we argue, was to constrain variability, especially in the outcomes but also the exposures, thereby limiting our ability to detect the expected socioeconomic gradient in the CVD outcomes and hypothesized associations between exposure to racial discrimination and the CVD outcomes, were such underlying causal relationships to exist.

In brief, null findings can occur for two very different reasons – above and beyond such well-established reasons as non-differential or differential measurement error, variables wrongly omitted from or included in the analytic models, or inadequate sample size [58], [59]. The first is that the hypothesis can be correct, but the postulated association is not observable in the examined study population [60]–[63]; for example, as famously observed by Geoffrey Rose [60], if everyone smokes, no association will be detected between smoking and lung cancer, despite their causal connection. The second is that the hypothesis is incorrect, such that the association would not be observed even with the ideal data set [60]–[63]. Determining which explanation is most plausible consequently requires careful scrutiny of study participants' range of exposures and outcomes [60]–[63].

In the case of our study, given plausible theorized pathways by which racial discrimination and its social and economic consequences can become embodied and harm cardiovascular health (e.g., direct physiological damage due to chronic activation of stress responses; health-harming behavioral responses, e.g., smoking; adverse social and biophysical exposures associated with residential and occupational segregation and material deprivation; and inadequate medical care) [1]–[10], we suggest two lines of evidence support our interpretation of our null findings. The first and most important is that we observed unexpectedly small socioeconomic and racial/ethnic differences in risk for the study outcomes, despite a large body of population-based research documenting substantial socioeconomic and racial/ethnic (and especially US black vs. white) inequities in cardiovascular mortality, disease, and risk factors [4]–[6], [64], [65]. Additionally, the two psychometrically-validated explicit racial discrimination measures we used have been associated – in many studies of more economically diverse populations – with risk of diverse health outcomes, including CVD risk factors [1], [3], [4]–[6], [66]. The combination of the unexpectedly poor health status of both the black and white participants, along with recruiting participants during the 2008–2010 economic recession (perhaps linked to the poorer socioeconomic profile of the white participants compared to populations in Boston, the US, and other studies and their use of community health centers), could thus conceivably account for not only the null findings vis a vis racial discrimination and the selected health outcomes, but also lack of a black excess risk for the Framingham CVD 10 year risk score and also the lack of impact of controlling for socioeconomic position on the observed black excess risk for SBP and hypertension.

Ascertaining whether or not the novel implicit and structural measures we employed accurately capture the relevant exposures, however, will require more empirical investigation, given the paucity of analogous research. Of note, the IAT methodology itself has been extensively validated [25], [67], [68], and we have shown that the novel IAT measures we have developed are immune, as expected, to social desirability (i.e., saying what one thinks is the expected “right” answer, as opposed to what one believes [11], [25], [69]), whereas lower self-reports of racial discrimination were strongly associated with higher social desirability [11]. Also suggesting the Jim Crow measure may have promise, the handful of extant epidemiologic studies [70]–[74] on the health impact of Jim Crow indicate its abolition was associated with declines in infant mortality and preventable mortality among adults, with likely intergenerational effects [7]. Underscoring the measure's potential importance, in 2013 all US-born persons whose birth year was in or preceded 1964, i.e., age 47 and older, were born when Jim Crow was legal with the implication that their parents and grandparents, if also US-born, were likewise exposed, depending on their race/ethnicity, to the discrimination or benefits conferred by Jim Crow [1], [7], [74]. Suggesting too that further work is needed to understand the strength and limitations of the explicit measures we used [20], [24], ours is not the first study to find an effect for one explicit measure not detected with another measure; new results from the Jackson Heart Study (4939 African American participants, ages 35–84), for example, found a weak positive association between hypertension and lifetime racial discrimination (as measured by the EOD; OR  = 1.08 (95% CI 1.02, 1.15), comparing the highest vs. lowest quartile, adjusted for age and gender), but no associations with the EDS [75].

No published studies, moreover, have investigated associations between racial discrimination and the Framingham CVD 10-year risk score, even as US investigations have documented socioeconomic and racial/ethnic (including black vs. white) disparities in this and related Framingham risk scores [76]. Although validated for use across diverse US racial/ethnic groups [12], concerns exist as to whether these scores may underpredict risk of adverse CVD outcomes among persons subjected to economic deprivation [64]. Keeping this caveat in mind (which would lead to conservative estimates of effect), integrated measures of CVD risk (whether the Framingham scores or analogous measures [12], [13], [77]) could nevertheless be useful, precisely because they provide a clinically-relevant global estimate of risk that takes into account the varying levels of the specified key risk factors [12], [13], [77].

In summary, results of our study underscore why appraisal of findings on racial discrimination and health, and by extension any exposure-outcome association, requires careful contextualization of study participants and their range of exposures and outcomes [1], [7]. If our interpretation is correct, it has important implications for research on health inequities, with regard to clarifying circumstances in which associations arising from causal relationships, if extant, may or may not be observed.

## Supporting Information

Table S1 **Univariate and multivariable associations of racial discrimination, socioeconomic position, and additional covariates with cardiovascular outcomes:** ***My Body My Story*** **study (504 black, 501 white US born non-Hispanic participants; Boston, 2009–2010) (imputed data).**
(DOCX)
