# Supplementary material for: Racial Discrimination & Cardiovascular Disease Risk: My Body My Story Study of 1005 US-Born Black and White Community Health Center Participants (US)
Source: PLoS One. 2013 Oct 18;8(10):e77174. doi: 10.1371/journal.pone.0077174 (PMC3799698; doi:10.1371/journal.pone.0077174)
Supplement: Table S1 — Univariate and multivariable associations of racial discrimination, socioeconomic position, and additional covariates with cardiovascular outcomes: My Body My Story study (504 black, 501 white US born non-Hispanic participants; Boston, 2009–2010) (imputed data). (DOCX) [file pone.0077174.s001.docx]

| **Table S1: Univariate and multivariable associations of racial discrimination, socioeconomic position, and additional covariates with cardiovascular outcomes: *My Body My Story* study (504 black, 501 white US born non-Hispanic participants; Boston, 2009-2010) (imputed data).** | | | | | | | | | | | | |
| --- | --- | --- | --- | --- | --- | --- | --- | --- | --- | --- | --- | --- |
| **Variable** | **Systolic blood pressure (mm Hg)** | | | | **Hypertension** | | | | **Framingham CVD 10-yr risk score**  **(log-transformed values)** | | | |
|  | **β (95% CI)** | | | | **OR (95% CI)** | | | | **β (95% CI)** | | | |
|  | **Black** | | **White** | | **Black** | | **White** | | **Black** | | **White** | |
|  | **Univariate** | **Multivariable** | **Univariate** | **Multivariable** | **Univariate** | **Multivariable** | **Univariate** | **Multivariable** | **Univariate** | **Multivariable** | **Univariate** | **Multivariable** |
| ***Racial discrimination*** |  |  |  |  |  |  |  |  |  |  |  |  |
| **Explicit** |  |  |  |  |  |  |  |  |  |  |  |  |
| EOD: continuous (0-9) | -0.21 (-0.74, 0.32) | -0.31 (-0.87, 0.24) | -0.09 (-0.92, 0.73) | -0.38 (-1.14, 0.39) | 0.96 (0.90, 1.02) | 0.94 (0.86, 1.02) | 1.04 (0.93, 1.15) | 1.02 (0.90,1.16) | ***0.04 (0.01, 0.07)*** | -0.01 (-0.03, 0.01) | 0.03 (-0.02, 0.08) | 0.00 (-0.03, 0.03) |
| **Implicit** |  |  |  |  |  |  |  |  |  |  |  |  |
| IAT: black vs. white (B/W) as target | 0.40 (-4.00, 4.81) | 1.43 (-4.05, 6.90) | -1.12 (-4.82, 2.57) | -3.40 (-7.27, 0.47) | 1.00 (0.57, 1.73) | 0.86 (0.39, 1.90) | 1.03 (0.64, 1.65) | 1.04 (0.54,1.99) | -0.09 (-0.38, 0.20) | -0.17 (-0.38, 0.05) | -0.02 (-0.26, 0.21) | -0.01 (-0.17, 0.14) |
| IAT:  me vs. them (M/T) as target | -1.96 (-5.86, 1.93) | 0.72 (-4.49, 5.92) | ***-4.86 (-9.08, -0.64)*** | -3.26 (-7.24, 0.72) | 0.75 (0.46, 1.25) | 1.03 (0.47, 2.27) | 0.77 (0.45, 1.32) | 0.82 (0.42,1.62) | -0.20 (-0.45, 0.06) | -0.03 (-0.24, 0.19) | ***-0.36 (-0.63,-0.08)*** | ***-0.17 (-0.33, -0.01)*** |
| Interaction: IAT B/W x IAT M/T |  | -3.51 (-15.7, 8.67) |  | 7.22 (-3.14, 17.58) |  | 1.05 (0.17, 6.51) |  | 1.49 (0.30, 7.57) |  | 0.15 (-0.35, 0.65) |  | -0.13 (-0.52, 0.27) |
| **Structural** |  |  |  |  |  |  |  |  |  |  |  |  |
| born in Jim Crow state: yes vs. no (ref) | 2.18 (-0.86, 5.23) | -1.26 (-4.66, 2.15) | 2.85 (-3.16, 8.87) | 2.76 (-2.73, 8.24) | ***1.92 (1.28, 2.89)*** | 0.97 (0.59, 1.60) | 0.62 (0.27, 1.44) | 0.64 (0.25, 1.69) | ***0.57 (0.38, 0.76)*** | -0.08 (-0.22, 0.06) | -0.08 (-0.49, 0.33) | 0.22 (-0.01, 0.44) |
| ***Sociodemographic*** |  |  |  |  |  |  |  |  |  |  |  |  |
| Age (years) | ***0.39 (0.22, 0.57)*** | ***0.30 (0.10, 0.51)*** | ***0.32 (0.14, 0.49)*** | ***0.18 (0.00, 0.35)*** | ***1.09 (1.07, 1.12)*** | ***1.10 (1.06, 1.13)*** | ***1.06 (1.04, 1.09)*** | ***1.07 (1.04, 1.11)*** | ***0.08 (0.07, 0.09)*** | ***0.08 (0.07, 0.09)*** | ***0.07 (0.06, 0.08)*** | ***0.07 (0.06, 0.07)*** |
| Gender: men vs women (ref) | 2.56 (-0.49, 5.61) | ***3.52 (0.10, 6.93)*** | ***10.72 (7.94, 13.49)*** | ***9.78 (6.69, 12.87)*** | 1.24 (0.84, 1.83) | 1.42 (0.85, 2.37) | ***1.95 (1.34, 2.83)*** | ***1.78 (1.06, 2.99)*** | ***0.93 (0.75, 1.11)*** | ***0.82 (0.68, 0.95)*** | ***1.09 (0.93, 1.25)*** | ***1.03 (0.91, 1.15)*** |
| ***Economic*** |  |  |  |  |  |  |  |  |  |  |  |  |
| Poverty:  <200% vs.  >=200% (ref) | 1.13 (-1.76, 4.03) | 0.84 (-2.10, 3.77) | -0.17 (-3.09, 2.75) | -0.00 (-2.70, 2.69) | 1.44 (0.97, 2.13) | 1.50 (0.92, 2.45) | 0.90 (0.61, 1.32) | 0.69 (0.43, 1.10) | 0.05 (-0.13, 0.24) | 0.02 (-0.10, 0.15) | 0.11 (-0.08, 0.30) | -0.04 (-0.15, 0.07) |
| Education:  < high school  (HS) | -0.12 (-5.16, 4.92) | -1.74 (-7.23, 3.75) | 4.03 (-1.13, 9.19) | 1.00 (-4.25, 6.26) | 1.72 (0.87, 3.38) | 0.93 (0.40, 2.12) | ***1.96 (1.02, 3.75)*** | 0.96 (0.41, 2.26) | ***0.33 (0.01, 0.65)*** | -0.08 (-0.29, 0.14) | ***0.74 (0.41, 1.07)*** | 0.11 (-0.11, 0.32) |
| >= HS and  < 4 yrs college | -1.28 (-5.23, 2.67) | -0.46 (-4.42, 3.50) | 2.74 (-0.32, 5.80) | 0.91 (-2.34, 4.17) | 0.79 (0.48, 1.31) | 0.64 (0.35, 1.16) | 1.45 (0.97, 2.18) | 0.95 (0.55, 1.66) | 0.02 (-0.23, 0.27) | -0.07 (-0.23, 0.08) | ***0.52 (0.32, 0.71)*** | ***0.18 (0.05, 0.30)*** |
| >=4 yrs coll (ref) | 0.0 | 0.0 | 0.0 | 0.0 | 1.0 | 1.0 | 1.0 | 1.0 | 0.0 | 0.0 | 0.0 | 0.0 |
| Census tract poverty:  >=20 % | -1.08 (-7.07, 4.91) | -1.27 (-6.90, 4.36) | -1.08 (-5.39, 3.23) | -2.45 (-6.33, 1.42) | 1.39 (0.69, 2.80) | 1.35 (0.61, 3.01) | 1.40 (0.77, 2.56) | 1.19 (0.58, 2.45) | 0.13 (-0.24, 0.51) | -0.02 (-0.22, 0.19) | 0.20 (-0.10, 0.49) | 0.06 (-0.10, 0.23) |
| >=5% and  <20% | -1.40 (-7.34, 4.55) | -1.23 (-6.88, 4.42) | -2.16 (-5.99, 1.68) | -2.14 (-5.58, 1.31) | 1.27 (0.62, 2.59) | 1.13 (0.49, 2.59) | 1.25 (0.74, 2.11) | 1.42 (0.76, 2.65) | 0.07 (-0.31, 0.44) | -0.06 (-0.28, 0.16) | -0.00 (-0.26, 0.25) | 0.01 (-0.13, 0.15) |
| < 5% (ref) | 0.0 | 0.0 | 0.0 | 0.0 | 1.0 | 1.0 | 1.0 | 1.0 | 0.0 | 0.0 | 0.0 | 0.0 |
| Parents’/guardians’ highest education: <HS | 1.04 (-5.34, 7.41) | -0.19 (-6.78, 6.40) | 1.99 (-3.33, 7.30) | 1.23 (-3.38, 5.83) | ***1.96 (1.02, 3.77)*** | 1.49 (0.75, 2.97) | 1.98 (0.98, 4.01) | 1.99 (0.80, 4.94) | ***0.33 (0.00, 0.65)*** | 0.09 (-0.13, 0.32) | 0.28 (-0.09, 0.66) | 0.01 (-0.18, 0.21) |
| >= HS and  < 4 yrs college | -1.26 (-6.55, 4.03) | -1.17 (-5.92, 3.57) | 1.86 (-1.66, 5.39) | 1.93 (-1.22, 5.09) | 1.08 (0.58, 2.01) | 1.19 (0.68, 2.08) | 1.40 (0.92, 2.14) | 1.38 (0.83, 2.28) | -0.04 (-0.38, 0.30) | 0.04 (-0.12, 0.21) | 0.21 (-0.03, 0.44) | 0.06 (-0.06, 0.18) |
| >=4 yrs coll (ref) | 0.0 | 0.0 | 0.0 | 0.0 | 1.0 | 0.0 | 1.0 | 1.0 | 0.0 | 0.0 | 0.0 | 0.0 |
| ***Psychosocial*** |  |  |  |  |  |  |  |  |  |  |  |  |
| Response to unfair treatment: act/quiet | 2.24 (-2.68, 7.16) | 0.84 (-4.23, 5.91) | 3.65 (-1.16, 8.46) | -1.13 (-5.61, 3.35) | 1.37 (0.72, 2.63) | 1.03 (0.49, 2.20) | 1.66 (0.91, 3.04) | 1.35 (0.65, 2.81) | 0.14 (-0.19, 0.46) | 0.06 (-0.15, 0.28) | 0.27 (-0.06, 0.59) | -0.05 (-0.23, 0.13) |
| accept./talk | -0.41 (-4.43, 3.61) | -1.29 (-5.30, 2.72) | 2.64 (-1.24, 6.52) | 0.62 (-2.86, 4.10) | 1.40 (0.83, 2.35) | 1.14 (0.62, 2.10) | 1.61 (0.98, 2.63) | ***1.86 (1.05, 3.31)*** | 0.16 (-0.10, 0.42) | 0.09 (-0.07, 0.24) | 0.15 (-0.10, 0.40) | ***0.16 (0.03, 0.30)*** |
| accept/quiet | -1.91 (-7.26, 3.44) | -2.95 (-8.25, 2.35) | 3.08 (-1.83, 7.99) | -0.93 (-5.36, 3.50) | 1.34 (0.67, 2.67) | 1.23 (0.56, 2.70) | 1.11 (0.58, 2.10) | 0.68 (0.32, 1.45) | -0.04 (-0.39, 0.30) | -0.02 (-0.23, 0.19) | ***0.42 (0.11, 0.73)*** | -0.06 (-0.23, 0.11) |
| act/talk (ref) | 0.0 | 0.0 | 0.0 | 0.0 | 1.0 | 1.0 | 1.0 | 1.0 | 0.0 | 0.0 | 0.0 | 0.0 |
| Social desirability  (per 10 units) | 0.01 (-0.46, 0.48) | 0.03  (-0.44, 0.50) | 0.17 (-0.32, 0.65) | 0.29 (-0.16, 0.74) | 1.01 (0.95, 1.07) | 1.01 (0.94, 1.08) | 1.02 (0.96, 1.08) | 1.03 (0.95, 1.11) | -0.01 (-0.03, 0.02) | 0.02  (-0.00, 0.03) | 0.01 (-0.02, 0.04) | 0.01 (-0.00, 0.03) |
| ***Anthropometric*** |  |  |  |  |  |  |  |  |  |  |  |  |
| BMI (kg/m^2^) | ***0.29 (0.10, 0.48)*** | ***0.26 (0.06, 0.46)*** | ***0.75 (0.55, 0.94)*** | ***0.66 (0.46, 0.87)*** | ***1.05 (1.02, 1.08)*** | ***1.06 (1.03, 1.09)*** | ***1.11 (1.08, 1.15)*** | ***1.12 (1.08, 1.17)*** | 0.01 (-0.01, 0.02) | ***0.02 (0.02, 0.03)*** | ***0.05 (0.04, 0.06)*** | ***0.04 (0.03, 0.05)*** |
| Waist-to-hip ratio * 100 | ***0.26 (0.06, 0.47)*** | 0.07 (-0.15, 0.28) | ***0.52 (0.33, 0.71)*** | 0.07 (-0.13, 0.27) | ***1.04 (1.01, 1.07)*** | 1.02 (0.99, 1.06) | ***1.06 (1.03, 1.09)*** | 1.03 (0.99, 1.07) | ***0.04 (0.03, 0.06)*** | ***0.01 (0.01, 0.02)*** | ***0.05 (0.04, 0.06)*** | 0.01 (-0.00, 0.01) |
| ***Health behavior*** |  |  |  |  |  |  |  |  |  |  |  |  |
| Cigarette smoking: |  |  |  |  |  |  |  |  |  |  |  |  |
| Current &  smoked within  8 hrs of exam | ***-3.80 (-7.19, -0.40)*** | -3.26 (-6.69, 0.17) | -2.79 (-6.65, 1.06) | -1.89 (-5.50, 1.72) | 0.80 (0.52, 1.22) | 0.76 (0.46, 1.28) | 1.17 (0.71, 1.92) | 1.54 (0.82, 2.87) | ***0.52 (0.31, 0.74)*** | ***0.40 (0.26, 0.54)*** | ***0.40 (0.15, 0.65)*** | ***0.46 (0.32, 0.61)*** |
| Current & did  not smoke with-  in 8 hrs of exam | 0.60 (-5.35, 6.55) | 1.38 (-4.30, 7.05) | 1.24 (-4.24, 6.71) | 2.17 (-2.87, 7.21) | 1.24 (0.62, 2.50) | 1.40 (0.64, 3.07) | 1.80 (0.90, 3.58) | ***2.61 (1.09, 6.24)*** | 0.29 (-0.03, 0.62) | ***0.26 (0.04, 0.48)*** | ***0.27 (-0.10, 0.64)*** | ***0.40 (0.20, 0.59)*** |
| Ex-smoker | -1.83 (-5.88, 2.23) | ***-4.68 (-8.73, -0.62)*** | -1.95 (-5.43, 1.53) | -2.12 (-5.23, 0.99) | 1.56 (0.90, 2.72) | 1.05 (0.55, 2.01) | 1.03 (0.65, 1.62) | 1.03 (0.60, 1.77) | ***0.44 (0.18, 0.69)*** | -0.05 (-0.21, 0.11) | -0.07 (-0.29, 0.15) | -0.12 (-0.24, 0.00) |
| Never smoker  (ref) | 0.0 | 0.0 | 0.0 | 0.0 | 1.0 | 1.0 | 1.0 | 1.0 | 0.0 | 0.0 | 0.0 | 0.0 |
| Alcohol within 8 hrs of exam: yes vs no (ref) | -0.05 (-3.66, 3.57) | -0.89 (-4.56, 2.79) | -3.36 (-6.83, 0.12) | ***-5.23 (-8.55, -1.91)*** | 1.03 (0.65, 1.64) | 0.81 (0.46, 1.42) | 0.98 (0.63, 1.54) | 0.62 (0.36, 1.07) | 0.00 (-0.22, 0.23) | 0.02 (-0.11, 0.16) | ***0.23 (0.00, 0.45)*** | -0.08 (-0.21, 0.04) |
| Food within 8 hrs of exam: yes vs no (ref) | 5.04 (-0.33, 10.41) | 3.49 (-1.43, 8.41) | 8.77 (-0.20, 17.75) | 6.21 (-1.54,13.96) | 2.01 (0.90, 4.51) | 2.05 (0.98, 4.29) | 1.99 (0.66, 5.98) | 1.52 (0.37, 6.19) | ***0.37 (0.05, 0.69)*** | 0.16 (-0.03, 0.35) | 0.22 (- 0.43,0.87) | -0.02 (-0.40, 0.36) |
| **Medication** |  |  |  |  |  |  |  |  |  |  |  |  |
| Taking anti-hypertensive medication: yes vs no (ref) | ***9.13 (6.40, 11.86)*** | ***7.13 (4.08, 10.19)*** | ***9.78 (6.54, 13.03)*** | ***4.34 (1.02, 7.65)*** |  |  |  |  |  |  |  |  |
